# Hijacking the Cellular Mail: Exosome Mediated Differentiation of Mesenchymal Stem Cells

**DOI:** 10.1155/2016/3808674

**Published:** 2016-01-06

**Authors:** Raghuvaran Narayanan, Chun-Chieh Huang, Sriram Ravindran

**Affiliations:** Department of Oral Biology, University of Illinois at Chicago, Chicago, IL 60612, USA

## Abstract

Bone transplantation is one of the most widely performed clinical procedures. Consequently, bone regeneration using mesenchymal stem cells and tissue engineering strategies is one of the most widely researched fields in regenerative medicine. Recent scientific consensus indicates that a biomimetic approach is required to achieve proper regeneration of any tissue. Exosomes are nanovesicles secreted by cells that act as messengers that influence cell fate. Although exosomal function has been studied with respect to cancer and immunology, the role of exosomes as inducers of stem cell differentiation has not been explored. We hypothesized that exosomes can be used as biomimetic tools for regenerative medicine. In this study we have explored the use of cell-generated exosomes as tools to induce lineage specific differentiation of stem cells. Our results indicate that proosteogenic exosomes isolated from cell cultures can induce lineage specific differentiation of naïve MSCs* in vitro *and* in vivo*. Additionally, exosomes can also bind to matrix proteins such as type I collagen and fibronectin enabling them to be tethered to biomaterials. Overall, the results from this study show the potential of cell derived exosomes in bone regenerative medicine and opens up new avenues for future research.

## 1. Introduction

The key to tissue regeneration is to achieve lineage specific differentiation of stem cells. Growth factors play a key role in inducing stem cell differentiation. However, from a tissue engineering perspective, they also pose the biggest challenge. The choice of growth factors to achieve a desired cellular response, the estimation of dosage and release mechanisms, and the associated complications with release profiles and kinetics are some of the biggest challenges that are limiting several tissue engineering approaches.

These issues can be resolved if stem cell differentiation can be achieved without the need for growth factor delivery systems. One strategy is to use extracellular matrix (ECM) derived biomaterials. We have published previously on the use of tissue-specific, cell derived ECM scaffolds for achieving lineage specific differentiation of stem cells [1–3]. Although such matrices are very promising candidates for regenerative medicine, their transition from bench to bedside is still riddled with regulatory hurdles that may take years to resolve. Therefore, the immediate clinical need is to generate products that can enhance the bioactivity of existing clinical materials. With respect to bone regenerative applications and in particular applications that require large volumes of bone to be regenerated, the immediate need is to augment the bioactivity of clinical materials such as allograft demineralized bone matrices (DBMs) and collagen membranes.

In recent years the use of microRNAs (miRNAs) to induce stem cell differentiation has gained popularity [4, 5]. miRNAs are noncoding RNAs that regulate posttranscriptional expression of target genes. miRNAs have the potential to be a successful tool in regenerative medicine. However, this field is relatively new and our knowledge of the range and dynamics of miRNAs and their roles in stem cell differentiation and also disease onset and progression is limited. Additionally, another challenge for the use of miRNAs in regenerative medicine is the development of safe delivery mechanisms. Although viral as well as nonviral mediated delivery of miRNAs has been studied, the most efficient mode of miRNA delivery is through the use of viral vectors [6]. The use of viral vectors is not a clinically viable solution in several cases [6]. Additionally, similar to growth factors, stem cells utilize several miRNAs at various stages of differentiation to achieve and maintain lineage specificity. Therefore, a more biomimetic approach is required.

In this regard the use of exosomes may offer a unique advantage. Exosomes are microvesicles that are generated by cells to facilitate intracellular communication [7]. Exosomes contain miRNAs and proteins that can induce a specific cellular response in target cells [8]. In the past 3-4 years, exosomes, especially mesenchymal stem cell (MSC) derived exosomes, have gained prominence in regenerative medicine research. Recent studies have shown that MSC derived exosomes behave as paracrine effectors and can be used to modulate immune response for inducing repair and regeneration of tissues such as kidney, heart, and nerve* in vivo* [9–12]. However, the use of exosomes to achieve lineage specific differentiation of stem cells has not been explored.

Published reports have shown a change in exosomal miRNA composition upon induction of MSC differentiation [13]. Additionally, exosomes can also be endocytosed by cells [14, 15]. We therefore hypothesized that the exosomes from osteogenic MSCs should be able to trigger the differentiation of naïve MSCs. In order to test this hypothesis, in this study, we have attempted the use of human marrow stromal cells (HMSCs) derived exosomes as agents to induce osteogenic differentiation of undifferentiated HMSCs.

## 2. Materials and Methods

### 2.1. Cell Culture

Primary human marrow derived stromal cells (HMSCs) were used in this study. Cells were purchased from ATCC and cultured in growth media containing minimum essential medium alpha (*α*-MEM) (Gibco), 20% fetal bovine serum (FBS) (Gibco), 1% L-glutamine (Gibco), and 1% antibiotic-antimycotic solution (anti-anti, Gibco). For induction of osteogenic differentiation, osteogenic medium containing growth media supplemented with 100 *μ*g/mL ascorbic acid, 10 mM *β*-glycerophosphate, and 10 mM dexamethasone was used.

### 2.2. Isolation of Exosomes

HMSCs were seeded to confluence in 100 mm tissue culture dishes. They were then cultured 2 or 4 weeks as per experimental requirement in the presence of either growth or osteogenic differentiation media. The 2-week exosomes were used for only one experiment on HMSCs in 2D cultures. For all other experiments, exosomes generated from 4-week cultures were used. For collection of exosomes, the cells were placed in serum free (growth or differentiation) medium for a day. The exosomes were isolated from the collected serum free media using the ExoQuick-TC (System Biosciences) exosome isolation reagent as per protocol specified by the manufacturer. The isolated exosomes were suspended in PBS. Exosomes isolated from every 10 mL of media were resuspended in 250 *μ*L of PBS. Exosome suspensions were normalized to cell number from the tissue culture plate they were isolated from and diluted appropriately afterward to ensure that the amount of exosomes in a given volume is constant for samples obtained from different cells and batches. Cross-verification was performed by measuring RNA and total protein isolated from the exosome suspensions to ensure that RNA/protein concentration from the same volume of exosomes remained consistent.

### 2.3. Endocytosis of Exosomes

The RNA in the isolated exosomes was labeled using the Exo-Glow-Red labeling kit (System Biosciences) as per manufacturer instructions. 100,000 HMSCs were seeded on to glass coverslips and incubated with labeled exosomes or control solution (composition that went through the same labeling procedure but did not contain any exosomes) for 2 hours. The cells were then fixed in neutral buffered formalin and imaged using a Zeiss LSM 710 Meta confocal microscope. The cells were excited at 460 nm and the emission from the labeled exosomes was recorded at 650 nm.

### 2.4. *In Vitro* Differentiation

100,000 HMSCs were plated in 6-well tissue culture plates for 2D culture or embedded in 3D within collagen hydrogels generated from 250 *μ*L of 1 mg/mL type I collagen (BD Biosciences). They were then incubated for 48 hours with exosomes isolated from 500,000 cells or an equivalent volume of the similarly diluted isolation reagent. Exosomes were isolated from cells cultured for 4 weeks using growth as well as osteogenic differentiation media. Experiments were performed in triplicate. After specified time points, the RNA from the cells was isolated followed by cDNA synthesis. Quantitative real time RTPCR (qRT PCR) was performed to analyze the expression levels of genes representative of osteogenic differentiation of MSCs. Expression of 14 proosteogenic genes was analyzed.  Table 1 lists the genes and the primers used in this study. Data is presented as mean fold change with respect to control samples that did not contain exosomes but were treated similarly in every other way. Statistical significance is represented as *P* value calculated using Student's *t*-test.

Total protein was isolated from the 2D experiments. Equal amounts of protein were then subjected to SDS-PAGE, transferred on to nitrocellulose and subjected to immunoblotting using the following primary antibodies: tubulin (Sigma 1/10,000), BMP2 (Abcam 1/1000), TGF*β* (Abcam, 1/1000), and platelet derived growth factor (PDGF, Abcam, 1/1000). The blots were stained with fluorescent secondary antibodies (anti-rabbit 680 and anti-mouse 800 Licor, 1/15000) and imaged using a Licor Imager equipped with the manufacturer's imaging software.

### 2.5. Transmission Electron Microscopy (TEM)

TEM was used to verify the presence of exosomes in the purified samples and also to look for binding to type I collagen. For verification of exosome presence, 10 *μ*L of a 1 in 10 dilution of exosome samples was placed on to fomvar/carbon coated nickel TEM grids and incubated for 30 minutes. The grids were then washed extensively in double deionized water and dried. The grids were then stained using phosphotungstic acid as per standard procedures and imaged using a JOEL JEM-1220 TEM.

### 2.6. Type I Collagen Binding

Dose dependent binding of exosomes to type I collagen was analyzed using ELISA. 96-well assay plates were coated with 5 *μ*g of type I collagen per well. The coated plates were incubated for 1 hour at room temperature with increasing volumes of exosomes. The plates were then washed 3 times in PBS, fixed using 4% neutral buffered formalin, permeabilized using PBS containing 0.5% triton x-100, and blocked for 1 hour at room temperature with PBS containing 5% BSA. The wells were then incubated for 1 hour at room temperature with CD63 antibody (Abcam, 1/1000 dilution), washed 3 times with PBS, and incubated for 1 hour with HRP conjugated secondary antibody (1/3000 dilution). All antibody dilutions were performed in PBS containing 5% BSA. Turbo TMB ELISA substrate was used to for the colorimetric assay followed by addition of acid stop solution (1 M sulfuric acid). The absorbance at 495 nm was measured using a Bio-tek ELISA plate reader. The experiment was performed in quadruplicate. The absorbance was normalized to the control wells (no exosome added, but containing type I collagen and treated with both primary and secondary antibodies) and the results were plotted graphically with volume of exosome on the *x*-axis and normalized absorbance units on the *y*-axis.

### 2.7. Binding of Exosomes to the ECM

100,000 HMSCs were plated onto cover glass placed inside 6-well plates. After 48 hours of culture, the wells were decellularized as per previously published protocol [1, 16] leaving behind the cell-secreted ECM. The wells were then incubated with exosomes isolated from 500,000 cells or similarly diluted reagent for 1 hour at 37°C. The wells were then fixed in 4% neutral buffered formalin, permeabilized, and immunostaining was performed as per previously published protocols [16] using rabbit polyclonal fibronectin (1/100 dilution) and mouse monoclonal CD63 (1/100) antibodies followed by respective anti-rabbit (TRITC conjugated) and anti-mouse (FITC conjugated) secondary antibodies. The cover slips were mounted and imaged using a Zeiss LSM 710 Meta confocal microscope.

### 2.8. *In Vivo* Implantation of 3D Scaffolds

All animal experiments were performed in accordance with protocols approved by the UIC animal care committee (A3460-01). Exosomes isolated from 1.25 million cells (100 *μ*L suspension) were added to 1 cm × 1 cm clinical grade type I collagen membranes (Zimmer collagen tape). 250,000 HMSCs were then seeded on to the membranes. Note that the cell to exosome ratio was maintained constant for the* in vitro *and* in vivo* experiments. The membranes were then implanted subcutaneously on the back of immunocompromised athymic nude mice as per previously published protocols [2, 3] for 4 weeks. The membranes were then extracted, fixed in neutral buffered 4% formalin, embedded, and sectioned into 5 *μ*m thin sections. The sections were subjected to H&E, alizarin red, and von Kossa staining as per standard protocols. Fluorescence immunohistochemistry was performed using mouse monoclonal anti-phosphorylated serine, threonine, and tyrosine antibody (pSTT, Abcam, 1/100 dilution), mouse monoclonal anti-dentin matrix protein 1 (DMP1) antibody (a kind gift from Dr. Anne George, University of Illinois at Chicago College of Dentistry, 1/2000 dilution), mouse monoclonal anti-vascular endothelial growth factor (VEGF) antibody (Abcam, 1/250 dilution), and mouse monoclonal anti-bone morphogenetic protein 2 (BMP2) antibody (Abcam, 1/100 dilution). The fluorescently stained sections were imaged using a Zeiss LSM 710 Meta confocal microscope. All sections were imaged as 3D z-stacks and represented as reconstructed 3D images using the Zeiss Zen imaging software.

## 3. Results and Discussion

### 3.1. Endocytosis of Exosomes

**Figure 1:**
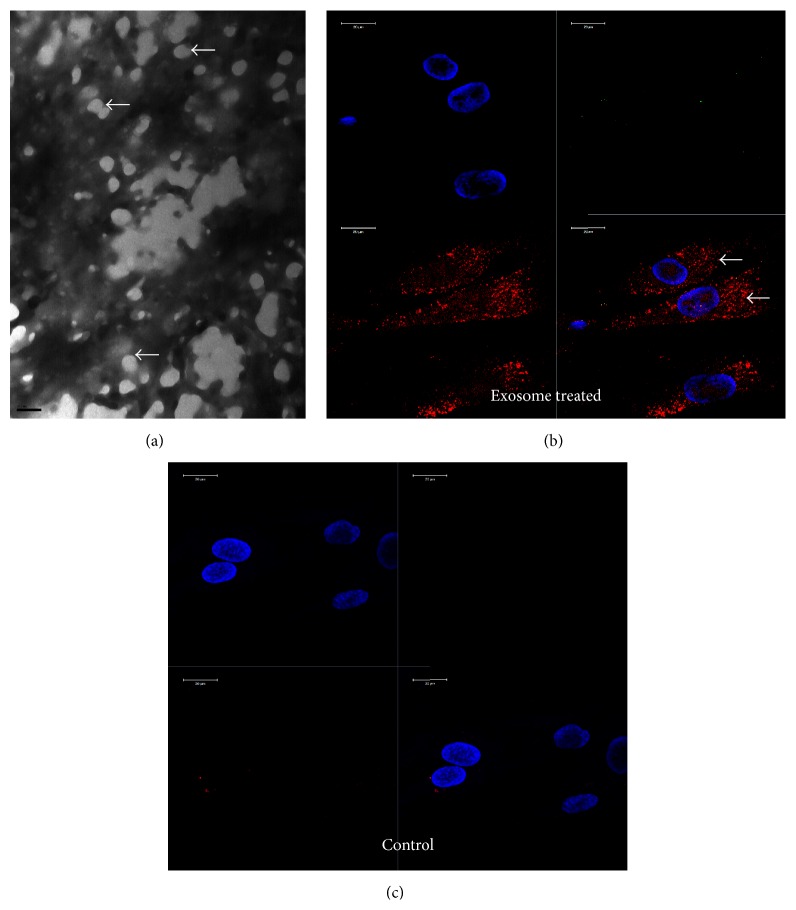
Generation and endocytosis of exosomes: (a) a representative TEM image showing the presence of exosomes in our purified samples. Scale bar represents 100 nm. The white arrows in the image point to exosomes. The grids were stained with phosphotungstic acid and hence the background is black. (b) Representative confocal micrograph showing the presence of endocytosed exosomes. White arrows point to the endocytosed exosomes. (c) Confocal micrograph showing the absence of exosomes or nonspecific presence of labeling dye. The scale bar in the confocal micrographs represents 20 *μ*m.

The presence of exosomes in the purified suspensions was verified by TEM (Figure 1(a)). In order for the exosomes to be effective as enhancers of differentiation, the key feature is the ability to be endocytosed by target cells. Published studies have shown endocytosis of tumor cell derived exosomes by both normal and oncogenic cells [8, 17]. We therefore proceeded to investigate if HMSC derived exosomes can be endocytosed by undifferentiated primary HMSCs.  Figure 1(b) shows that when 100,000 HMSCs were treated with labeled exosomes from 500,000 cells, the exosomes are endocytosed by the cells.  Figure 1(b) also shows transference of exosomal miRNA intracellularly as the labeling procedure uses acridine orange chemistry and labels the intra exosomal nucleic acids. On the other hand, a control preparation that went through the same labeling procedure but did not contain any exosomes did not show any intracellular presence (Figure 1(c)).

### 3.2. Exosome Mediated Differentiation of HMSCs* In Vitro* in 2D Cultures

Results presented in Figure 1 showed that exosomes could be endocytosed by HMSCs. We proceeded to investigate if the endocytosed exosomes can influence the fate of HMSCs by inducing cellular differentiation. Two different types of exosomes were used for these experiments: exosomes isolated from cells cultured under normal growth conditions (hereby referred to as regular exosomes) and exosomes isolated from cells cultured under osteogenic conditions (hereby referred to as osteogenic exosomes).  Table 2 shows the change in gene expression data when primary undifferentiated HMSCs were treated for 48 hours with regular and osteogenic exosomes isolated from 2-week cultures. Expression of 14 genes representative of induction of osteogenic differentiation was analyzed by qRT PCR. Only those that showed statistically significant change are represented in the table. Results presented in Table 2 show that the exosomes triggered an increase in the expression levels of growth factors bone morphogenetic protein 9 (BMP9) and transforming growth factor *β*1 (TGF*β*1). Both BMP9 and TGF*β*1 have been shown to be good inducers of osteogenic differentiation of MSCs [18, 19]. BMP9 is one of the most potent inducers of osteogenic differentiation and is more potent than BMP2 [20]. It was therefore encouraging to see that the exosomes could influence MSC differentiation. However, the change in the expression levels of transcription factors and ECM proteins required for osteogenic differentiation was modest but statistically significant.

**Figure 2:**
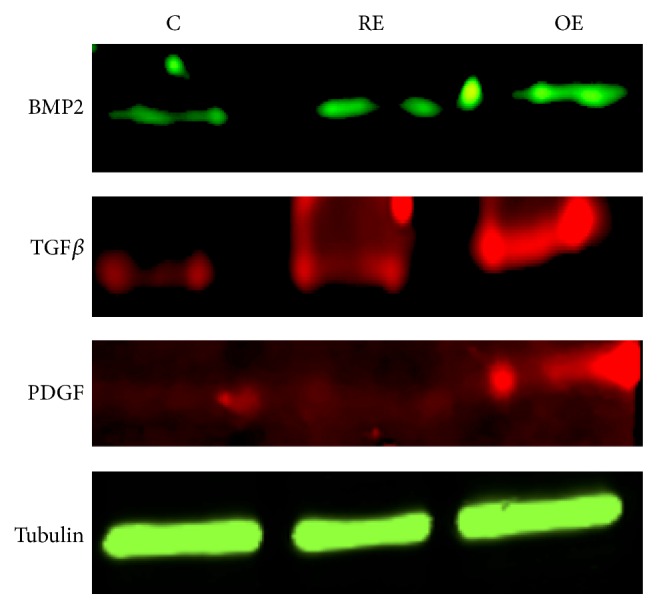
Immunoblotting experiments: images are representative immunoblots of BMP2, TGF*β*, PDGF, and tubulin (top to bottom). Note the increase in expression of the growth factors between control and exosome treated samples. Tubulin was used as loading control.

We then proceeded to investigate if exosomes from cultures under the influence of osteogenic medium for 4 weeks would generate a better response from undifferentiated HMSCs in terms of inducing osteogenic differentiation. Results presented in Table 3 show that both regular and osteogenic exosomes from 4-week cultures induced a very robust and statistically significant upregulation in several genes spanning growth factors, transcription factors, and ECM molecules. Although the osteogenic exosomes performed better than regular exosomes, we were surprised at the ability of regular exosomes to induce such a big change. HMSCs are known to undergo osteogenic differentiation when cultured for long periods at high confluence. We hypothesize that this change could be a result of a confluent culture of HMSCs undergoing differentiation and thereby generating exosomes with the potential to induce osteogenic differentiation. The protein expressions of growth factors BMP2, TGF*β*, and PDGF were verified by immunoblotting. Results presented in Figure 2 show that there was an increase in the protein expression levels of all the three proteins in the cells treated with exosomes. For these experiments, tubulin was used as the loading control.

### 3.3. Binding of Exosomes to ECM Proteins

Results presented thus far indicate the ability of exosomes to influence MSC differentiation. However, if exosomes are to be used as agents to induce lineage specific differentiation, they need to be tethered to the ECM so that they can be made available to the cells. Additionally, they should be accessible and effective when the MSCs are present in 3D matrices that simulate* in vivo *conditions.

**Figure 3:**
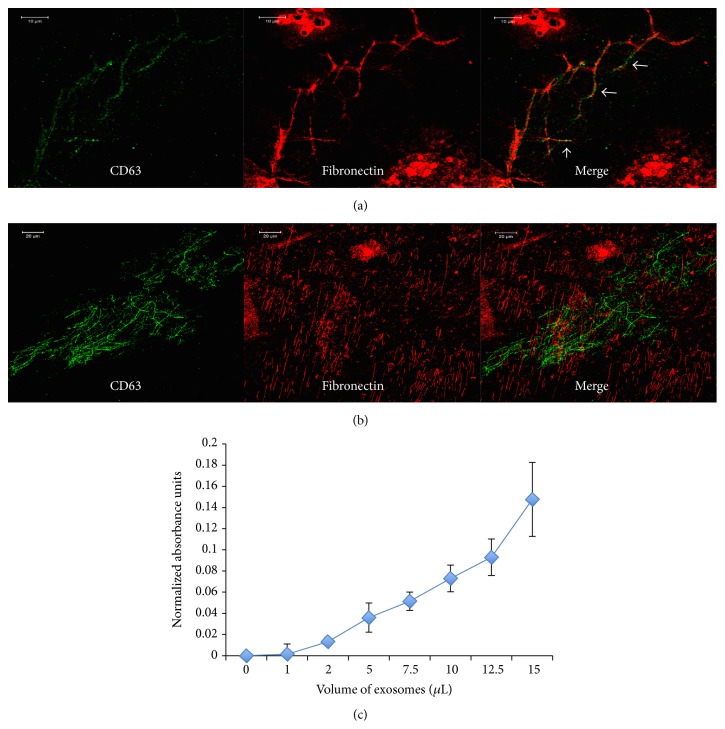
Binding of exosomes to ECM proteins: (a) confocal micrograph showing colocalization of exosomes (immunolabeled with CD63 antibody in green) with fibronectin (immunolabeled in red) present in the ECM of HMSCs. Arrows point to areas of colocalization. Scale bar represents 10 *μ*m. (b) Confocal micrograph showing exosomes immunolabeled with CD63 antibody bound to the ECM of HMSCs. Note the fibrillar pattern of binding indicating binding to an ECM structural protein. However, no colocalization was observed with fibronectin (immunolabeled in red) indicating the ability of the exosomes to bind multiple ECM proteins. Scale bar represents 20 *μ*m. (c) Graphical representation of dose dependent exosomal binding to type I collagen.

Exosomes are vesicles that pinch off from the plasma membrane. Therefore, the exosomal membrane is also composed of plasma membrane. Cells attach to ECM proteins using integrins and other cell surface receptors present on the plasma membrane. We therefore tested if exosomes can bind to the ECM secreted by HMSCs. Results presented in Figures 3(a) and 3(b) show that when HMSC-generated ECM is treated with exosomes, binding of the exosomes to ECM proteins is observed.  Figure 3(a) shows a representative confocal micrograph of HMSC derived exosomes (immunolabeled with CD63 antibody in green) bound to fibronectin (immunolabeled in red). The white arrows in the merged image of Figure 3(a) show areas of colocalization. On the other hand, the confocal image in Figure 3(b) shows exosomal presence in a fibrillar form representing binding to an ECM protein. However, no colocalization was observed with fibronectin. Additionally, no secondary antibody mediated nonspecific fluorescence was observed in the controls. Taken together, these results indicate that the exosomes can bind to multiple ECM proteins.

We quantitatively analyzed exosome binding to type I collagen by means of ELISA. Results presented in Figure 3(c) show a dose response curve indicating binding of exosomes (measured using CD63 antibody) to type I collagen-coated plates. Although we see an increase in bound exosomes with increase in dosage, we were not able to observe saturation. We hypothesize that, with the amount of surface area available to bind on type I collagen fibrils, the amount of exosomes required to saturate binding may be very high. Additionally, several plasma membrane integrins bind to type I collagen. Therefore, the binding curve observed could be a result of multiple integrins binding to type I collagen from different exosomes. Nevertheless, the experiment showed that exosomes could bind to type I collagen and that the amount of bound exosomes increased in a dose dependent manner.

### 3.4. Exosome Mediated Differentiation of HMSCs* In Vitro* in 3D Cultures

Having observed exosome mediated MSC differentiation and exosome binding to type I collagen, we proceeded to investigate if 4-week exosomes can be used to induce osteogenic differentiation of HMSCs cultured within type I collagen hydrogels in 3D. Results presented in Table 4 indicate change in gene expression of proosteogenic genes when HMSCs in 3D collagen hydrogels were cultured in the presence of exosomes. As before, the effect of regular and osteogenic exosomes was investigated. Results showed that both regular and osteogenic exosomes induced significant upregulation of proosteogenic genes. However, unlike the experiment performed on 2D cultures, the difference between the two types of exosomes was not as pronounced when the HMSCs were cultured in a 3D environment. Significant upregulation of growth factors, transcription factors, and ECM proteins was observed. Importantly, runx2 and Osterix, the two most important transcription factors for induction of osteogenic differentiation and osteogenesis, were significantly upregulated by both regular and osteogenic exosomes.

Collectively, these results indicate the potential of using cell-generated exosomes as differentiating agents to induce lineage specific differentiation of MSCs.

### 3.5. Exosome Mediated Differentiation of HMSCs* In Vivo*

Our next step in the evaluation of exosomes as differentiating agents was* in vivo *evaluation. For this purpose, we used clinical grade collagen membranes that are available commercially, as scaffolds for carrying HMSCs and exosomes. The membranes were wetted with the 4-week exosome solution and HMSCs were then seeded on to the membranes and implanted subcutaneously on the back of athymic nude mice for 4 weeks as described under Section 2. Sections from the explants were subjected to histology and immunohistochemistry.

**Figure 4:**
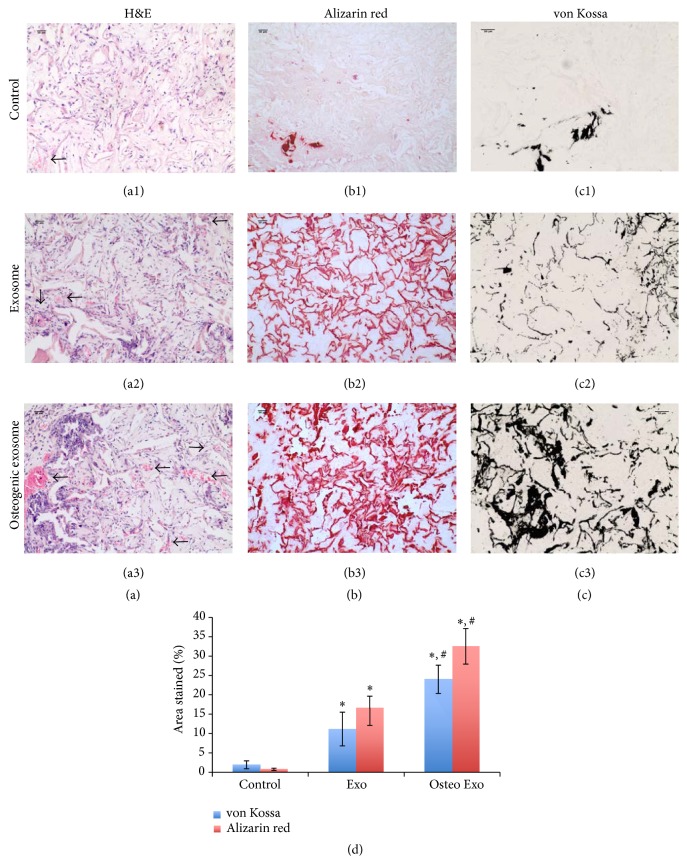
Histology of scaffold explant sections from* in vivo *implantation: (a1, a2, and a3) Representative H&E stained images of sections from control, regular exosome treated, and osteogenic exosome treated scaffolds containing HMSCs, respectively. Arrows point to blood vessels. Note the increase in the presence of blood vessels in the images from exosome treated scaffolds. (b1, b2, and b3) Representative alizarin red stained images of sections from control, regular exosome treated, and osteogenic exosome treated scaffolds containing HMSCs, respectively. Note the robust increase in calcium presence in exosome treated sample sections. (c1, c2, and c3) Representative von Kossa stained images of sections from control, regular exosome treated, and osteogenic exosome treated scaffolds containing HMSCs, respectively. Note the increase in presence of calcium phosphate in the exosome treated sections. Also, note the increase in vascularization and calcium phosphate presence in sections from osteogenic exosome treated samples compared to regular exosome treated samples. (d) is a graphical representation of histological data from triplicate experiments showing mean percentage area stained with von Kossa (blue bars) and alizarin red (red bars) stains. Error bars represent SD. *∗* represents statistical significance with respect to control (*P* < 0.01). # represents statistical significance between exosome and osteogenic exosome treated groups (*P* < 0.05).

Results presented in Figures 4(a1), 4(a2), and 4(a3) show representative images of H&E stained sections from control, regular, and osteogenic exosome containing scaffold explants. The arrows in these images point to capillaries and blood vessels within the scaffolds indicating vascularization. Results indicate that the scaffolds containing exosomes showed more robust vascularization than the control scaffolds. Additionally, the scaffolds containing osteogenic exosomes had the best vascularization with the presence of large blood vessels (Figure 4(a3)).

Images presented in Figures 3(b) and 3(c) represent alizarin red (Figures 4(b1), 4(b2), and 4(b3)) and von Kossa (Figures 4(c1), 4(c2), and 4(c3)) stained sections, respectively, from the scaffold explants. When taken together, the images show that HMSCs seeded in scaffolds containing regular and osteogenic exosomes induced more robust calcium phosphate nucleation when compared to those seeded on control scaffolds. Additionally, increased calcium and phosphorus presence was observed in the scaffolds containing HMSCs and osteogenic exosomes when compared to the other two groups indicating that the osteogenic exosomes were more efficient in inducing osteogenic differentiation of the HMSCs followed by matrix mineralization. Figure 4(d) is a quantitation of histological data. We analyzed the percentage area stained in the micrographs of the explant sections stained with von Kossa and alizarin red dyes. Results showed that exosome treated sections showed significantly more percentage area stained compared to controls. Additionally, osteogenic exosome treated explant sections showed significantly more staining compared to regular exosome treated explant sections.

**Figure 5:**
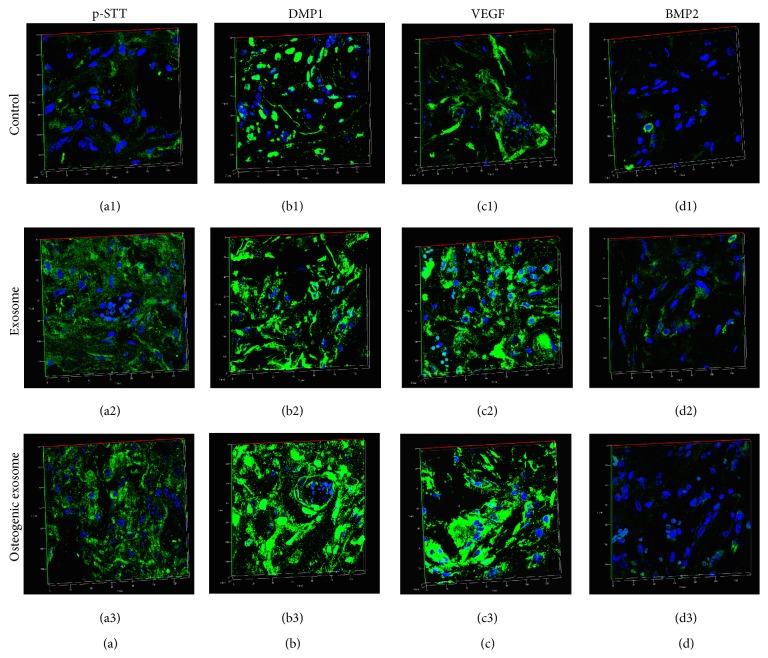
IHC of scaffold explant sections from* in vivo *implantation: all images are representative 3D reconstructions of z-stack confocal image slices. (a1, a2, and a3) represent sections from control, regular exosome, and osteogenic exosome treated samples, respectively, stained with pSTT antibody. (b1, b2, and b3) represent sections from control, regular exosome, and osteogenic exosome treated samples, respectively, stained with DMP1 antibody. (c1, c2, and c3) represent sections from control, regular exosome, and osteogenic exosome treated samples, respectively, stained with VEGF antibody. (d1, d2, and d3) represent sections from control, regular exosome, and osteogenic exosome treated samples, respectively, stained with BMP2 antibody. Except for BMP2, note the increase in the expression levels of proteins in the exosome treated samples.

Fluorescence IHC was performed on the explant sections to analyze the expression levels of proteins involved in matrix mineralization, vascularization, and osteogenic differentiation.  Figure 5 shows the results from these experiments. Figures show representative images of 3D reconstructions of z-stack confocal images of control (Figures 5(a1), 5(b1), 5(c1), and 5(d1)), regular exosome (Figures 5(a2), 5(b2), 5(c2), and 5(d2)), and osteogenic exosome (Figures 4(a3), 4(b3), 4(c3), and 4(d3)) treated scaffold sections.

Results indicate that the sections from scaffolds containing exosomes showed increased presence of phosphorylated proteins (pSTT staining in Figure 5(a)) as evidenced by increased presence of phosphorylated serine, threonine, and tyrosine residues in these sections (Figures 5(a2) and 5(a3) in comparison with Figure 5(a1)). Phosphorylated proteins serve as the source of phosphorus during calcium phosphate nucleation* in vivo*. Therefore, increased presence of these proteins indicates a higher potential for matrix mineralization. Another important protein involved in matrix mineralization is DMP1. Our results indicate an increase in DMP1 expression in sections from scaffolds containing exosomes compared to control scaffolds (Figures 5(b2) and 5(b3) in comparison with Figure 5(b1)).

We also observed an increase in the expression levels of VEGF in the exosome treated samples compared to controls (Figures 5(c2) and 5(c3) in comparison with Figure 5(c1)) indicating the potential of the exosome treated scaffolds to induce better vascularization. However, BMP2 protein expression remained low and constant among all samples. We hypothesize that BMP2, being a growth factor involved in osteogenic differentiation, may be required at earlier stages of differentiation and hence may not be expressed in high amounts during matrix mineralization phase. The sections are from scaffolds subjected to 4 weeks of* in vivo* implantation and judging by the protein expression and histology data, they are representative of the matrix mineralization phase. No secondary nonspecific fluorescence was observed in secondary antibody controls.

Overall, from the* in vivo* implantation experiments, our results indicate that both regular and osteogenic exosomes have the potential to induce osteogenic differentiation of naïve HMSCs. However, based on the histology data the osteogenic exosomes induce a more robust calcium deposition and calcium phosphate nucleation.

## 4. Conclusion

Bone is the second most transplanted organ in the human body [21]. Bone grafting is a procedure that is performed by orthopedic surgeons, oral and maxillofacial surgeons, dentists, and periodontists. In dentistry, a significant portion of people that require implants need bone graft surgeries before implant placement. With respect to children, over 75% of birth defects are craniofacial anomalies (such as cleft palate) that require bone reconstruction procedures [22]. Finally, with the wars in Iraq and Afghanistan, the incidence of injuries requiring significant bone reconstruction is at an all-time high.

Clinically, the gold standard for bone regenerative procedures is autografts. In cases that require significant amounts of bone, donor site morbidity becomes an issue when autografts are used. Under these circumstances, allograft bone is used. However, the osteoinductive as well as osteogenic capacity of allograft DBM is significantly lesser than autografts [21]. In some cases, FDA approved growth factors such as recombinant BMP2 are used to augment bone growth. Although it is very potent, dosage issues and ectopic effects are major problems facing BMP2 usage. Many complications have been reported recently causing serious safety concerns among clinicians [23, 24]. Therefore, the immediate need for improvement of clinical outcomes is a technique to enhance the effectiveness and predictability of bone regenerative procedures.

In this study, we present results that show that exosomes can be used to induce stem cell differentiation. Our results indicate that exosomes from osteogenic HMSCs can trigger lineage specific differentiation of undifferentiated HMSCs both* in vitro *and* in vivo*. Results also show that exosomes can bind to ECM proteins such as type I collagen and fibronectin. Based on these results, we predict that such exosomes can be used to either pretreat autologous cell populations prior to usage in patients or be tethered to compounds such as collagen membranes and DBM that are used clinically and contain type I collagen and fibronectin (in the case of DBM). Additionally, from a futuristic perspective, the exosomes can also be used in conjunction with decellularized biomimetic scaffolds to augment their performance. In addition, any new biomaterial that is developed to favor cell adhesion can be functionalized with cell type specific exosomes to induce lineage specific differentiation.

To our knowledge, this is the first report that shows the osteogenic potential of exosomes in regenerative medicine. Coupled with other characteristics such as immunomodulation, cell survival enhancement, and prevention of cell death [25], exosomes could serve as a powerful tool in regenerative medicine. However, additional studies that focus on the functions of individual exosomal miRNAs responsible for different aspects of the cellular response are needed to understand the overall functionality of exosomes. We believe that this information can also be used in the future to generate modified exosomes that overexpress certain important miRNAs that can augment the exosome potential manifold. Overall, we believe that, through this paper, we have unlocked a new biomimetic tool for regenerative medicine that capitalizes the functionality of nature's most potent intercellular communication tools.

## Figures and Tables

**Table 1:** List of primers used in this study for qRT PCR.

Gene	Forward	Reverse
FGF2	5′-AGA AGA GCG ACC CTC ACA TCA-3′	5′-CGG TTA GCA CAC ACT CCT TTG-3′
BMP2	5′-ACT ACC AGA AAC GAG TGG GAA-3′	5′-GCA TCT GTT CTC GGA AAA CCT-3′
GDF10	5′-AGA TCG TTC GTC CAT CCA ACC-3′	5′-GGG AGT TCA TCT TAT CGG GAA CA-3′
PHEX	5′-GAG GCA CTC GAA TTG CCC T-3′	5′-ACT CCT GTT TAG CTT GGA GAC TT-3′
ALPL	5′-ACT GGT ACT CAG ACA ACG AGA T-3′	5′-ACG TCA ATG TCC CTG ATG TTA TG-3′
TGFB1	5′-CAA TTC CTG GCG ATA CCT CAG-3′	5′-GCA CAA CTC CGG TGA CAT CAA-3′
RUNX2	5′-TGG TTA CTG TCA TGG CGG GTA-3′	5′-TCT CAG ATC GTT GAA CCT TGC TA-3′
OSX	5′-CCT CTG CGG GAC TCA ACA AC-3′	5′-AGC CCA TTA GTG CTT GTA AAG G-3′
OCN	5′-AGC CCA TTA GTG CTT GTA AAG G-3′	5′-CCC TCC TGC TTG GAC ACA AAG-3′
OPN	5′-GAA GTT TCG CAG ACC TGA CAT-3′	5′-GTA TGC ACC ATT CAA CTC CTC G-3′
VEGFA	5′-AGG GCA GAA TCA TCA CGA AGT-3′	5′-AGG GTC TCG ATT GGA TGG CA-3′
COL1	5′-GAG GGC CAA GAC GAA GAC ATC-3′	5′-CAG ATC ACG TCA TCG CAC AAC-3′
BMP9	5′-AGA ACG TGA AGG TGG ATT TCC-3′	5′-CGC ACA ATG TTG GAC GCT G-3′
BMP6	5′-TGT TGG ACA CCC GTG TAG TAT-3′	5′-AAC CCA CAG ATT GCT AGT GGC-3′
GAPDH	5′-CAG GGC TGC TTT TAA CTC TGG-3′	5′-TGG GTG GAA TCA TAT TGG AAC A-3′
B2M	5′-GAG GCT ATC CAG CGT ACT CCA-3′	5′-CGG CAG GCA TAC TCA TCT TTT-3′

**Table 2:** Two-week exosome mediated change in gene expression: 2D HMSC cultures.

Gene	HMSC regular exosomes fold change (*P* value)	HMSC Osteogenic exosomes fold change (*P* value)
Growth factors		
BMP9	16.62 (0.01)	25.90 (0.02)
TGFB1	2.13 (0.04)	1.96 (0.04)
Transcription factors		
RUNX2	1.08 (0.08)	1.17 (0.17)
ECM proteins		
OCN	1.36 (0.06)	1.11 (0.27)
OPN	0.84 (0.21)	1.84 (0.01)

Data represent fold change in gene expression when regular and osteogenic exosomes isolated from 2-week cultures were incubated with HMSCs in 2D cultures for 48 hours. Data are presented as mean fold change in gene expression with respect to control. *P* value specified in brackets shows statistical significance with respect to control obtained by means of Student's *t*-test.

**Table 3:** Four-week exosome mediated change in gene expression: 2D HMSC cultures.

Gene	HMSC regular exosomes fold change (*P* value)	HMSC osteogenic exosomes fold change (*P* value)
Growth factors		
BMP2	1.80 (0.0572)	11.54 (0.0027)
GDF10	1.76 (0.2400)	18.84 (0.0107)
BMP9	6.48 (0.003753)	34.13 (0.0034)
VEGFA	1.32 (0.0355)	2.93 (0.0009)
BMP6	2.80 (0.0143)	13.61 (0.0047)
FGF2	4.98 (0.0013)	9.88 (0.0042)
Transcription factors		
RUNX2	1.07 (0.4330)	1.61 (0.0390)
OSX	3.42 (0.0041)	40.61 (0.0049)
ECM proteins		
ALPL	2.33 (0.1113)	12.42 (0.0080)
OPN	3.93 (0.0207)	8.03 (0.0212)
COL1	4.98 (0.0355)	10.01 (0.0264)

Data represent fold change in gene expression when regular and osteogenic exosomes isolated from 4-week cultures were incubated with HMSCs in 2D cultures for 48 hours. Data are presented as mean fold change in gene expression with respect to control. *P* value specified in brackets shows statistical significance with respect to control obtained by means of Student's *t*-test.

**Table 4:** Four-week exosome mediated change in gene expression: 3D HMSC cultures.

Gene	HMSC regular exosomes fold change (*P* value)	HMSC osteogenic exosomes fold change (*P* value)
Growth factors		
BMP2	38.26 (0.0089)	21.34 (0.0255)
BMP9	34.13 (0.0003)	66.52 (0.0001)
BMP6	19.82 (0.0010)	13.35 (0.0134)
VEGFA	33.53 (0.0105)	16.42 (0.0191)
FGF2	17.62 (0.0021)	8.85 (0.0046)
TGFB1	27.39 (0.0061)	19.77 (0.0147)
GDF10	19.42 (0.0023)	15.26 (0.0001)
Transcription factors		
RUNX2	17.12 (0.0004)	10.53 (0.010216)
OSX	33.84 (0.0003)	20.86 (0.000897)
ECM proteins		
OCN	10.86 (0.0298)	6.59 (0.0048)
ALPL	25.27 (0.0089)	19.28 (0.0051)
OPN	17.75 (2.54*E* − 05)	9.03 (0.002589)
COL1	20.80 (0.0001)	12.04 (0.0063)

Data represent fold change in gene expression when HMSCs were cultured in 3D type I collagen hydrogels in the presence of regular and osteogenic exosomes isolated from 4-week cultures. Data are presented as mean fold change in gene expression with respect to control. *P* value specified in brackets shows statistical significance with respect to control obtained by means of Student's *t*-test.
